# Atrial fibrillation: a geriatric perspective on the 2020 ESC guidelines

**DOI:** 10.1007/s41999-021-00537-w

**Published:** 2021-11-02

**Authors:** M. Cristina Polidori, Mariana Alves, Gulistan Bahat, Anne Sophie Boureau, Serdar Ozkok, Roman Pfister, Alberto Pilotto, Nicola Veronese, Mario Bo, on behalf of the Special Interest Group “Cardiovascular Diseases” of the EuGMS

**Affiliations:** 1 Ageing Clinical Research, Department II of Internal Medicine and Center for Molecular Medicine Cologne, University of Cologne, Faculty of Medicine and University Hospital Cologne, Kerpener Str. 62, 50937 Cologne, Germany; 2 Cologne Excellence Cluster On Cellular Stress-Responses in Aging-Associated Diseases (CECAD), University of Cologne, Faculty of Medicine and University Hospital Cologne, Cologne, Germany; 3 Serviço de Medicina III, Hospital Pulido Valente, CHULN, Lisbon, Portugal; 4 Department of Internal Medicine, Division of Geriatrics, Istanbul Medical School, Istanbul University, Capa, 34390 Istanbul, Turkey; 5 Department of Geriatrics, CHU Nantes and Université de Nantes, CNRS, INSERM, l’Institut du Thorax, 44000 Nantes, France; 6 Department of Cardiology, University of Cologne, Faculty of Medicine and University Hospital Cologne, Cologne, Germany; 7 Department of Geriatric Care, Orthogeriatrics and Rehabilitation, Galliera Hospital, Genoa, Italy; 8 Department of Interdisciplinary Medicine, University of Bari, Bari, Italy; 9 Geriatric Unit, Department of Internal Medicine and Geriatrics, University of Palermo, Palermo, Italy; 10 Section of Geriatrics, Department of Medical Sciences, University of Turin, A.O.U. Città della Salute e della Scienza, Molinette, Corso Bramante 88, 10126 Turin, Italy; 11 Laboratory of Clinical Pharmacology and Therapeutics, Faculdade de Medicina, Universidade de Lisboa, Lisbon, Portugal; 12 Instituto de Medicina Molecular, Faculdade de Medicina, Universidade de Lisboa, Lisbon, Portugal

**Keywords:** Atrial fibrillation, Advanced age, Older patients, Anticoagulation, Cognitive impairment

## Abstract

**Background:**

The Task Force for the diagnosis and management of atrial fibrillation (AF) of the European Society of Cardiology (ESC) published in 2020 the updated *Guidelines for the Diagnosis and Management of Atrial Fibrillation with the contribution of the European Heart Rhythm Association (EHRA) of the ESC and the European Association for Cardiothoracic Surgery (EACTS)*.

**Methods and results:**

In this narrative viewpoint, we approach AF from the perspective of aging medicine and try to provide the readers with information usually neglected in clinical routine, mainly due to the fact that while the large majority of AF patients in real life are older, frail and cognitively impaired, these are mostly excluded from clinical trials, and physicians’ attitudes often prevail over standardized algorithms.

**Conclusions:**

On the basis of existing evidence, (1) opportunistic AF screening by pulse palpation or ECG rhythm strip is cost-effective, and (2) whereas advanced chronological age by itself is not a contraindication to AF treatment, a Comprehensive Geriatric Assessment (CGA) including frailty, cognitive impairment, falls and bleeding risk may assist in clinical decision making to provide the best individualized treatment.

## Key Summary Points

**Aim:**

To provide a geriatric perspective on the 2020 ESC Guidelines for the diagnosis and management of atrial fibrillation developed in collaboration with the European Association of Cardio–Thoracic Surgery (EACTS).

**Findings:**

While the large majority of AF patients in real life are older, frail and cognitively impaired, these are mostly excluded from clinical trials, and physicians’ attitudes often prevail over standardized algorithms. On the basis of existing evidence, we suggest that (1) opportunistic AF screening by pulse palpation or ECG rhythm strip is cost-effective, and (2) whereas advanced chronological age by itself is not a contraindication to AF treatment, a Comprehesive Geriatric Assessment (CGA) including frailty, cognitive impairment, falls and bleeding risk may assist in clinical decision making to provide the best individualized treatment.

**Message:**

The integration of CGA might positively influence clinical decision making in older patients with atrial fibrillation.

## Introduction

The Task Force for the diagnosis and management of atrial fibrillation (AF) of the European Society of Cardiology (ESC) published in 2020 the updated Guidelines for the Diagnosis and Management of Atrial Fibrillation with the contribution of the European Heart Rhythm Association (EHRA) of the ESC and the European Association for Cardiothoracic Surgery (EACTS) [1]. As the authors state in the preamble “(…) The complexity of AF requires a multifaceted, holistic and multidisciplinary approach to the management of AF… with the goal to further improve the structured management of AF patients, promote patient values, and finally improve patient outcomes… the Task Force includes cardiologists with varying subspecialty expertise, cardiac surgeons, methodologists and specialist nurses amongst its members (…).”. Reflecting the composition of the panel*,* this holistic “ABC strategy (A: anticoagulation/avoid stroke, B: better symptom control, C: detection and management of cardiovascular risk factors and concomitant diseases)” includes several developments with respect to the 2016 ESC guidelines [2], mainly focused on drug therapies (Table 1).

**Table 1:**
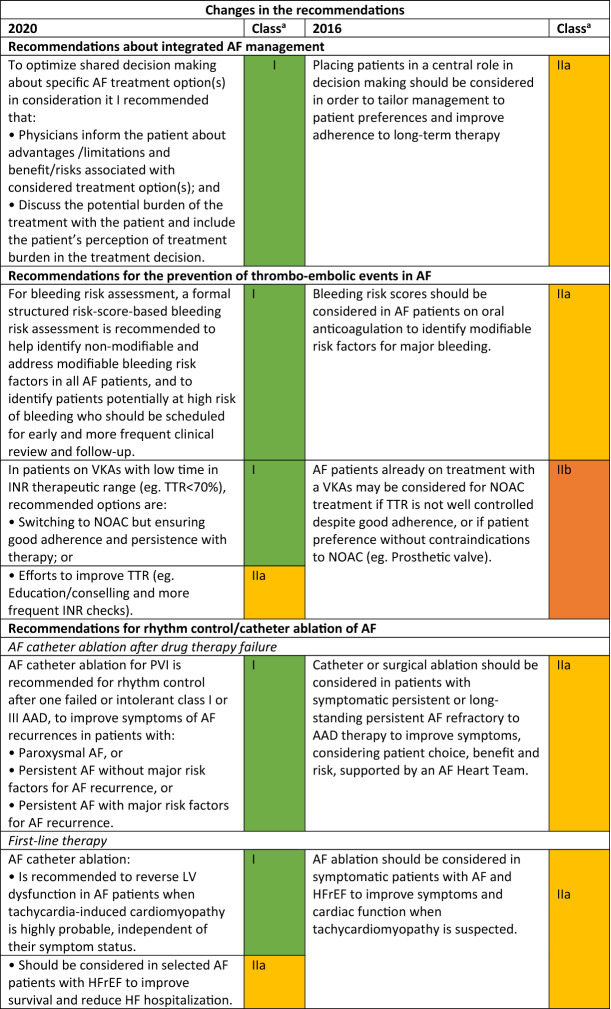

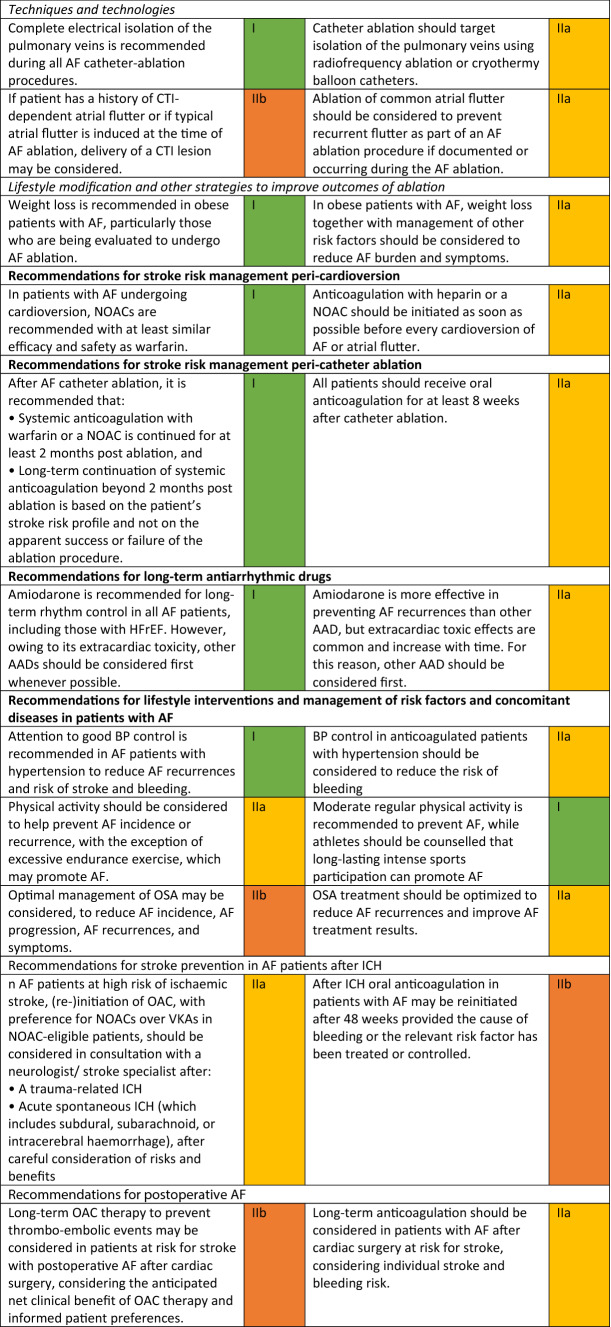
Changes in the ESC recommendations on atrial fibrillation, 2016–2020 [1, 2]. Mod. from [2]

*AAD* antiarrhythmic drug, *AF* atrial fibrillation, *BP* blood pressure, *CTI* cavotricuspid isthmus, *HFrEF* heart failure with reduced ejection fraction, *ICH* intracranial haemorrhage, *INR* international normalized ratio, *LV* left ventricular, *LVEF* left ventricular ejection fraction, *NOAC* non-vitamin K antagonist oral anticoagulant, *OAC* oral anticoagulant or oral anticoagulation, *PVI* pulmonary vein isolation, *TTR* time in therapeutic range, *VKA* vitamin K antagonist

^a^Class of recommendation

While the critical role of the ESC guidelines for the management of AF is out of the question, from a geriatric perspective such “holistic approach” comes short to address the complexity of older AF patients. Due to the nature of the aging process, in fact, there are crucial characteristics of older patients that, if neglected, often undermine, sometimes nullify the most perfect organ-centered treatment plan. These include multifactoriality, heterogeneity, atypical disease presentation, geriatric cascade, frailty just to mention a few. The appropriate use of the tools of geriatric medicine allows to balance the net clinical benefit of screening and therapeutic decisions and might be most appropriately shared and used in a meaningful interdisciplinary comanagement. On behalf of the Special Interest Group "Cardiovascular Diseases" of the European Geriatric Medicine Society (EuGMS), the aim of this overview is the disentanglement of age-related complexity by discussing specific questions arising in advanced age such as screening for AF, interplay with frailty, cognitive impairment, functional loss and falls. Furthermore, physicians’ attitudes and uncertainties in prescribing oral anticoagulant therapy (OAT) will be addressed.

## Atrial fibrillation in advanced age

Age is an independent risk factor for the development of AF [3, 4]. In 2010, the number of older individuals (≥ 75 years old) with AF in the European Union was estimated to be 5.6 million, but this is expected to double to 13.8 million by 2060, when subjects aged 80+ years will represent 65.2% of total AF cases in European Union [1, 5]. The relationship of AF with increased risk of thromboembolism, stroke and mortality is well-established [3]. Patients with AF exhibit increased hospitalizations due to stroke, heart failure, need for pacemaker implantation and adverse effects related to anticoagulant and antiarrhythmic therapy. In addition, there are other known specific, but often overlooked problems closely related to AF in this population, including cognitive impairment with and without dementia, frailty, decline in physical performance and loss of independence [6–8].

“Older people” include a very heterogeneous group of individuals, varying from independent and robust persons to dependent and very frail ones [9]. Age-related physiologic changes (low body mass index, altered body composition of muscle and fatty tissue), multimorbidity and polypharmacy (with high frequency of renal impairment, altered pharmacokinetic profile of drugs and high risk of drug–drug interactions), frailty, cognitive impairment and functional limitations and life-expectancy are factors that should be taken into account in clinical decision making [9]. In such a complex population, not suitable to general recommendations, the correct clinical approach is essential to offer the best medical care and, at the same time, to avoid harm and futility [8–10]. Although guidance exists for inclusion of older patients in clinical trials [11], these are still underrepresented in randomized clinical trials (RCTs), whereas the most robust, independent and cognitively intact older patients fit are usually enrolled [12]. Although the Comprehensive Geriatric Assessment (CGA) and frailty measurement tools have been recommended to help guide decisions about AF anticoagulation in older patients [13, 14], this is not generalized in clinical trials nor current practice yet.

As a matter of fact, older patients represent the largest majority of those with AF [1]. Notwithstanding, most recommendations, including the recent 2020 ESC guidelines for AF management, stem from RCTs poorly representative of real-world older people, and come short to deal with the complex health characteristics of these patients [15].

## Challenges of atrial fibrillation in real life

### Screening

In addition to increasing prevalence of clinical AF with advancing age, a significant number of older individuals do not show any symptoms despite going through an episode of AF [16]. Unfortunately, asymptomatic (silent) AF is independently associated with stroke and mortality to a similar extent compared to symptomatic AF [17]. It is estimated that 1.3% of the population aged ≥ 65 have undiagnosed, largely asymptomatic AF [18]. Patients with incidentally detected AF treated with OAT have stroke rates similar to matched individuals without AF [19]. Furthermore, their adjusted stroke and death rates are reduced, compared with those who are not treated or are treated with aspirin alone [19]. This provides justification for AF screening in high-risk populations. Of note, the 2020 ESC Guideline for AF management strongly recommends opportunistic screening of AF by pulse taking or ECG rhythm strip in patients ≥ 65 years of age [1].

A screening program is accepted as efficient if it shows high sensitivity and can be carried out with low costs and risks. There are several screening tools in use for AF detection which are quite comparable in terms of their sensitivity, specificity and ease of application. Pulse palpation is a traditional and proven to be effective method for AF screening in older adults [20]. Oscillometric blood pressure monitors and smartphone applications reported to have higher sensitivity and specificity values, although the validation studies are conducted in small number of participants [21, 22]. There are also single-lead handheld devices that provide an ECG strip, which offers an advantage of confirmation of diagnosis with an ECG recording. These devices also reported to be very effective and have been widely used in AF screening studies [23, 24]. Wearable continuous ECG monitors have been demonstrated to be well tolerated and to increase AF detection tenfold, compared with oscillometric screening with a BP monitor [25].

All of these screening methods seem advantageous in older adults in terms of being practical, as patients remain seated, pass through brief measurements and do not need to undress. However, they also bring some disadvantages. Pulse palpation is the tool of choice for opportunistic screening due to its high sensitivity and cost effectiveness. Unfortunately, pulse is infrequently assessed in routine care. Cardiac auscultation can be the other option for detecting irregular heartbeats, but it is also less frequently preferred. There are several applications in use, however, most of them are not clinically validated. In addition, some of them require a noise-free trace for optimal performance. Furthermore, although they provide high sensitivity, their specificity is lower which can cause anxiety, create extra work and cost verifying diagnosis with an ECG [26]. Although the interest of the older population in technology has increased in recent years, the rate of use of smart phones or other devices with advancing age has not been proven yet. This makes the self-initiating, telemedical or wearable-based screening of AF currently not realistic, especially in oldest-old, cognitively impaired and very frail individuals. Hence, the optimum choice of AF screening tool is still a debate and mostly depends on patient profile and screening setting.

Another controversy regarding AF screening in older adults is which strategy of screening is more effective. According to a recent meta-analysis, there was no statistically significant difference between opportunistic (offered as part of a routine medical evaluation) and systematic (general or targeted screening of a high-risk population) screening [27]; yet, opportunistic screening is likely to be more cost-effective [28]. Settings that have been used effectively include community-based and others based in primary care, specialist practices, general or specialist clinics or pharmacies [29]. Indeed, primary care physicians have the potential to be at the forefront in screening programs. One of the biggest challenges about the settings is ensuring the link for referral to confirm diagnosis and establish proper work-up. In terms of providing a direct link with diagnosis, treatment and follow-up, primary care and outpatient clinics seem the most advantageous. In fact, health resources vary widely between countries and health systems. Therefore, the setting and strategy for screening should be country and health-system specific [29].

In this context, the best available evidence suggests that opportunistic screening of AF in older population is cost-effective and should be performed. Older AF patients without cognitive impairment can be educated for checking their pulses intermittently, as self-detection of AF through pulse palpation was shown feasible in older adults [30]. Annual events (like influenza vaccination) can be a good opportunity for screening in this particular population [31, 32]. The introduction of health checks including AF screening in general practice, and obligation of presenting practice reports (like the General Practice contract developed in 1990 in England) [33], may increase detection of AF. In addition to primary health care workers, staff working in secondary and tertiary health care units dealing with older adults, and also nursing home staff should be educated for importance of AF detection and ECG interpretation in a routine basis. In addition to opportunistic screening, systematic screening programs for patients aged ≥ 75 years should be considered, since a significant number of this age group are vulnerable to AF outcomes due to their comorbidities, frailty, and cognitive impairments [1].

### Patients’ global health status

Patients with AF in clinical practice are older, have higher disease burden and are more frequently affected by functional limitations and dementia than those enrolled in RCTs [34]. Moreover, direct oral anticoagulants (DOACs) trials as well as observational studies did not consider geriatric syndromes such as frailty, cognitive impairment and functional dependence, which have been demonstrated to influence physicians’ decision about DOACs use in older persons [34]. Therefore, beyond conventional embolic and bleeding risk scores, a decision-making guidance that incorporates factors such as frailty and dementia would be more helpful in determining the therapeutic approach. Indeed frailty, dementia and disability are among the most commonly cited reasons for OAT under-prescription, although evidence to support this decision is controversial [4].

#### Frailty

It has been reported that cardiologists subjectively identify frailty in the presence of problems in motility, cognition, malnutrition and sarcopenia [35]. In this context, frail and frailty are terms frequently used to label some older person on the basis of a subjective mix of disease burden, poor health status and cognitive or functional impairment. In the wide armamentarium of frailty tools, there are two basic conceptualizations of frailty. The Cardiovascular Health Study (CHS)-derived frailty “phenotype” identifies a sarcopenia-dependent model of frailty, which is diagnosed when at least three of five criteria among slow gait speed, low physical activity, unintentional weight loss, self-reported exhaustion and muscle weakness are recognized. In the seminal paper by Fried et al. this frailty phenotype was demonstrated to be associated with several adverse clinical outcomes, including worsening mobility and disability, hospitalizations and mortality over 7 years in community-dwelling older persons [36]. In recent years, several other tools have been proposed to identify this frailty phenotype, including the Simplified Fried test [37], the Short Physical Performance Battery (SPPB) [38], the 5 m gait speed [39]*,* the Study of Osteoporotic Fractures (SOF) index [40, 41], the simple Frail Scale [42] and the SARC-F questionnaire [43]. A quite different approach to frailty conceptualization was developed by Rockwood et al. with the Canadian Study of Health and Aging (CSHA)-derived Frailty Index (FI) [44]. This is a 70-item form based on the accumulation of deficits (including functional limitations and disabilities, cognitive and sensory impairment, psychosocial variables and number of diseases), and its score is associated with increased probability of short-term risk of institutionalization, mortality and hospitalization. These authors further developed the seven-point Clinical Frailty Scale (CFS, a semi-quantitative eye-ball global judgement of frailty or vulnerability), which proved to be correlated with the FI and associated with increased risk of mortality and institutionalization [45]. The Multidimensional Prognostic Index (MPI) [46] (which is derived by a formal CGA through the inclusion of functional cognitive, nutritional and comorbidity scores and social support network) has also been demonstrated to be predictive of mortality and adverse clinical outcomes [47, 48].

The ESC guidelines for the diagnosis and management of AF (section 11.13) [1] state that “frailty, comorbidities, and increased risk of falls do not outweigh the benefits of OAT”. However, despite we can concur with this simple statement, OAT prescription in older AF patients yet remains a troublesome decision, and persisting uncertainties are well represented by the current underuse of appropriate OAT in frail older patients with AF, despite their increased risk of embolic stroke and death [15, 34, 49]. Wilkinson et al recently suggested that “The lack of evidence to guide optimal care for patients with AF and frailty might in part explain the gap between current guidelines and clinical practice in management of these patients” [7].

Chronological age per se is not an acceptable criterion to guide clinical decision making, as convincingly demonstrated during the SARS-Cov-2 pandemic [50]. As both the CGA and the MPI are time-demanding procedures in daily clinical practice, it is difficult to implement them systematically in cardiology or general internal wards. In the quest for an easier alternative to this complex assessment, “frailty” appeared as a captivating surrogate. However, the growing number of frailty tools available, and the limited consensus on how to define and measure this complex state have generated confusion in several clinical settings, including the persistent uncertainties around the clinical benefit of OAT in frail older patients. We argue that most of these uncertainties might originate from the different clinical implications linked to the adoption of different frailty tools. Indeed, albeit under the same definition of “frailty”, the two basic different conceptualizations (frailty phenotype vs accumulation of deficits) recognize different individuals and generate different clinical and prognostic implications. Whereas the CHS-frailty phenotype identifies patients at risk of adverse clinical outcomes in the presence of stressors [36], both the CFS and the FI recognize patients with global poor health status and with limited survival [36, 51–55]. Therefore, it is not surprising that using different tools to recognize “frailty” may identify older persons with rather different health status and residual life-span , thereby leading to discordant conclusions in many clinical settings, including AF [34, 56], and may generate confusion among expertise areas outside geriatrics.

Although a recent systematic review and meta-analysis on the management of AF for older persons with frailty concluded that “frailty is highly prevalent and associated with adverse clinical outcomes, and that there is a lack of evidence on the interaction of frailty and OAT with clinical outcomes to guide optimal care in this setting” [7], on the basis of current evidence it seems reasonable that older persons with AF and the “frail phenotype” might be considered for OAT, because of an expected net clinical benefit [34, 56–60], whereas at the moment there is scant, if any, evidence of benefit in those with severe frailty according to the FI/CFS, who are also more frequently denied OAT [7, 61, 62].

#### Cognitive impairment and falls

Within the frame of the increasing exploitation of the concept of multidimensional frailty [63], particular attention should be offered to the bidirectional effects of cognitive decline and AF. If, in fact, AF-related stroke predisposes to dementia, there is an association between AF and dementia independent of stroke which demands a great deal of attention when managing older patients. Usually, the focus of healthcare practitioners concentrates on new-onset dementia, as about one-third of all stroke patients develop the condition within 5 years and AF patients have a 2.7-fold dementia risk after stroke [64, 65]. Two meta-analyses revealed ≈ 30% increased risk of dementia in AF after adjustment for cerebrovascular events [65, 66]. Furthermore, AF is related to cognitive impairment or dementia in younger ages [67]. In these studies, no brain imaging was performed to rule out clinically silent strokes as the underlying pathophysiology. In a case-referent study, which included magnetic resonance imaging brain imaging, stroke-free individuals with AF showed difficulties in learning, memory, attention and executive function compared with healthy referents [68]. Nonischemic mechanisms include cerebral hypoperfusion, vascular inflammation and genetic factors. Cerebral hypoperfusion and hypoxia have been demonstrated to be associated with increasing AF burden, and may be further worsened by concomitant heart failure with reduced ejection fraction [69–71]. There is growing evidence that implementing use of DOACs and maintenance of sinus rhythm after cardioversion or catheter ablation, may reduce the risk of cognitive decline [72–75].

While the mechanisms of AF-related cognitive impairment are multiple, interrelated and strongly associated to age-related changes [76–78], their consideration is essential in the management of older AF patients. As a consequence, a cognitive screening should be part of the routine evaluation of AF patients, especially in advanced age. However, as in turn the diade cognitive impairment-AF in the context of the heart–brain syndrome is chacterized by a complex interaction of several interwoven factors—including functional performance, psychosocial aspects, multimorbidity and polypharmacy-, a multidomain screening of the patient followed by a CGA in comanagement with the geriatrician appears to be the best available option to disentangle complexity and implement patient-centered, goal-oriented, value-based care in real life [79, 80]. Within a targeted CGA, a formal cognitive assessment would be helpful in AF patients, and cognitive impairment at mild-to moderate stage should not be viewed as a general contraindication to OAT, especially if well-managed from a logistically point of view. On the contrary, the appropriate management of AF has been consistently shown to improve cognitive performance [72, 73]. Similarly to cognitive impairment, predisposition to falls is common in frail patients, and is often perceived as an important issue in starting OAT. Patients on OAT at high risk of falls do not consistently have a significantly increased risk of major bleedings [34]. While a multidimensional frailty assessment with screening of cognitive and gait/motoric functions is helpful to disclose important components of clinical decision making, current guidelines do not require fall risk estimation in candidates to OAT. As in the presence of cognitive impairment, the risk of fall should not be considered per se as a contraindication to the use of DOACs of general AF management. On the contrary, gait disturbances arising from cerebral blood perfusion and orthostatic deregulation might considerably benefit from prompt diagnosis and management of AF.

#### Comorbidities and bleeding risk

Older patients with AF are at risk of stroke and thromboembolism; therefore, planning a proper anticoagulant treatment soon after the diagnosis is essential. Unfortunately, advancing age is also associated with increased bleeding risk [81]. In this context, the bleeding risk (usually assessed through the HAS-BLED score) should not discourage physicians from prescribing OAT in patients with high risk of stroke, but rather prompt them to take action on controlling the modifiable bleeding risk factors and establishing a stricter and more frequent follow-up program [1]. Furthermore, these embolic and bleeding scores should be re-checked periodically in all AF patients [1].

Although previous bleeding events do not represent an absolute contraindication to OAT, there are several clinical situations which deserve a careful assessment in older AF patients. Older AF patients with spontaneous bleeding during OAT interruption, or unidentifiable or not treatable site of bleeding, or with recurrent bleeding from multiple angiodysplasias in the gastrointestinal tract, should be carefully assessed about the opportunity of withholding OAT [82]. Although several observational cohort studies suggest a survival and thromboembolic benefit in those who restarted OAT after major bleeding (MB) events [83, 84], clinicians should also consider that AF patients aged 80 years or older with a previous major gastrointestinal bleeding experience the greatest mortality (roughly 45% and 65% at 2 and 5 years, respectively) [85]; therefore, shared decision-making on the basis of the expected net clinical benefit is strongly advisable in these circumstances.

### Physicians’ attitudes in prescribing OAT

Prescription of OAT to older AF patients is a more complex clinical decision than a simple calculation of cardio-embolic and bleeding risk scores. Clinicians are well aware that some or most of their older AF patients may have limited residual life-span because of a relevant burden of pathologies and/or geriatric syndromes. Indeed, whereas in phase III DOAC trials all-cause mortality was 4.7%/year, with cardiac death contributing for 46% of deaths [86], real-world observational studies depict a different scenario. Among 186,461 Medicare beneficiaries (mean age 79.5 years) with AF, mortality was by far the most frequent major clinical events (occurring in 19.5% at 1 year and 48.8% at 5 years) [85]. Although several retrospective observational cohort studies demonstrated significant reduction of all-cause mortality in older patients treated with DOACs, regardless of poor health, functional conditions and multidimensional frailty, in most of these studies the mortality benefit was not accounted for by a significant reduction of stroke, suggesting that at least in part this mortality benefit might represent a selection bias (that is, OAT is prescribed to those perceived with longer survival). In this context, implementation of standardized tools described above to evaluate short-term mortality in older AF patients might assist clinicians to address this therapy to those who may derive some benefit in their residual life-span. The EUROSAF (European Study of Older Subjects with Atrial Fibrillation) project has begun to better accompany the daily clinical choice of managing OAT in older people [87]. EUROSAF is an observational study aimed at assessing the effectiveness and risks of anticoagulant therapy in frail older subjects with AF, stratified by the presence of frailty using the above described MPI [63, 87]. Some preliminary data suggest that almost half of these patients are not treated with OAT, particularly if they are frail according to the MPI values [88]. Future data regarding the effect of anticoagulants (newer and older) could be important to see if frailty can be a significant determinant in mortality and cardiovascular events in people treated or not with OAT. In this context, the role of the CGA and its derivatives with high prognostic value such as the MPI, seems to be a crucial driver in taking clinical decisions in the frame of practical algorithms to be used in frail multi-morbid and poly-treated older patients with AF.

Unfortunately, uncertainties do not finish when coming to the decision to prescribe OAT. The recent 2020 ESC guidelines recommend use of DOACs in preference to VKA, except for patients with prosthetic mechanical heart valves and moderate to severe mitral valve stenosis [1]. Several DOACs rankings [89–92] and expert opinions [93–96] have been published to assist physicians to select the DOAC according to individual patient’s characteristics. Apixaban has been demonstrated to have an excellent safety-efficacy balance, and suggested as a reasonable first choice either in older patients or in subjects with chronic renal failure [95]. The recently updated 2019 American Geriatrics Society Beers criteria recommend a cautious use of dabigatran and rivaroxaban in AF patients aged ≥ 75 years because of greater risk of gastrointestinal bleeding [97]. In a recent report from the Fit-fOR-The-Aged (FORTA) classification (evaluating benefit, risk and appropriateness of drugs for older patients in everyday clinical settings), Apixaban was labelled A among OATs, meaning it was seen as the drug with the most favorable risk/benefit ratio in older patients [98].

These medications are fixed-dose oral regimens available in two different dose options, which have been variously named (standard, full or higher dose, and reduced or lower dose); anyway, dose prescription should be in keeping with drug-specific dosing guidelines (Table 2). However, as some clinicians get into difficulties in prescribing OAT to these complex older patients, many others also struggle with using DOAC recommended doses in this population. Indeed, several studies from different settings and countries consistently reported high prevalence of inappropriate DOAC dosing, with inappropriate reduced dosing being largely prevalent, particularly in older patients [99–102].

**Table 2:** Dose adjustment of direct thrombin inhibitor and oral factor Xa inhibitors for stroke prevention in patients with nonvalvular atrial fibrillation and specific conditions, according to the European Medicines Agency summary of product characteristics

	Rivaroxaban≠	Apixaban¶	Edoxaban^	Dabigatran
Full dose	20 mg OD	5 mg BID	60 mg OD	150 mg BID
Age ≥ 80 years	20 mg OD	2.5 mg BID if another criterion*	60 mg OD	110 mg BID
Age 75–79 years	20 mg OD	5 mg BID	60 mg OD	110 mg BID for consideration
Body weight ≤ 60 kg	20 mg OD	2.5 mg BID if another criterion *	30 mg OD	–
SerCr ≥ 1.5 mg/l	–	2.5 mg BID if another criterion*	–	–
CrCl 30–49 ml/min	15 mg OD	5 mg BID	30 mg OD	110 mg BID for consideration
CrCl 15–29 ml/min	15 mg OD	2.5 mg BID	30 mg OD	Not recommended
CrCl < 15 ml/min	Not recommended	Not recommended	Not recommended	Not recommended
Concomitant therapy:				
Dronedarone	Not recommended	–	30 mg OD	Not recommended
Cyclosporine	–	–	30 mg OD	Not recommended
Erythromycin	20 mg OD	–	30 mg OD	–
Ketoconazole	Not recommended	Not recommended	30 mg OD	Not recommended
HIV protease inhibitors (e.g., ritonavir)	Not recommended	Not recommended	–	Not recommended
Verapamil	**–**	5 mg BID	60 mg OD	110 mg BID

*OD* once a day, *BID* two times a day, *CrCl* creatinine clearance, *HIV* human immunodeficiency virus

≠https://www.ema.europa.eu/en/medicines/human/EPAR/xarelto

^¶^https://www.ema.europa.eu/en/medicines/human/EPAR/eliquis

^https://www.ema.europa.eu/en/medicines/human/EPAR/lixiana

In a recent review about the pros and cons of inappropriate underdosing of oral Factor Xa activated Inhibitors (oFXaIs) [103] we demonstrated that although some underdosing may be ascribed to involuntary errors, a substantial proportion of it might reflect an intentional “cautious” approach to DOAC use in selected patients. Notably, very advanced age, female gender, presence of CKD, higher embolic and bleeding risk, previous bleeding or perceived high risk of bleeding, and concomitant use of antiplatelet drugs or nonsteroidal anti-inflammatory drugs (NSAIDs) were demonstrated to be associated with underdosing [103]. However, current evidence suggest that patients’ characteristics rather than OAT intensity are associated with the risk of bleeding events, as suggested by the observation that the rates of MB in RCTs were higher among patients treated with oFXaIs at reduced dose than in those receiving full dose [104]. Moreover, real-life studies did not demonstrate a net clinical benefit by inappropriate underdosing of oFXaIs, but rather an increased risk of adverse events, including hospitalizations for cardiovascular causes and stroke, without a significant reduction of bleeding events [103].

In this context, a potential novel therapeutic approach in older AF frail patients came from the recently published Edoxaban Low-Dose for Elder Care Atrial Fibrillation Patients (ELDERCARE-AF), which compared a once-daily 15-mg dose of edoxaban with placebo in 984 Japanese patients (mean age 86.6 years, 57.4% female) who had nonvalvular AF and in whom standard OAT was not recommended for at least one of the following reasons: a low creatinine clearance (15–30 ml per min), a history of bleeding from a critical area or organ or gastrointestinal bleeding, low body weight (≤ 45 kg), continuous use of NSAIDs, or current use of an antiplatelet drug [92]. Among the 681 patients who completed the trial the annualized rate of stroke or systemic embolism and MB were 2.3% vs 6.7% (*p* < 0.001), and 3.3% vs 1.8% (*p* = 0.09), in the edoxaban vs the placebo group, respectively, without substantial between-group difference in death from any cause [105]. These encouraging findings for a “humanitarian” OAT in the oldest patients should, however, be confirmed in other geographical areas and compared with appropriate DOAC doses before being considered an acceptable strategy in oldest patients.

Aspirin monotherapy was shown to be ineffective for stroke prevention in AF, as well as associated with increased risk of stroke in older patients [106]. Since the landmark BAFTA study [107], OAT (including warfarin and DOACs) has been consistently demonstrated to outweigh aspirin in terms of clinical net benefit in older AF patients [108–110]. The AVERROES study clearly demonstrated that is no evidence of clinical benefit from prescribing antiplatelet therapy compared with Apixaban to older AF patients [111], and current European recommendations strongly advice against this practice [1, 2], Likewise, use of dual antiplatelet therapy (DAPT) as an alternative to OAT is not recommended due to lower stroke prevention and similar bleeding rates [112].

Despite the increased bleeding risk, concomitant use of OAT and antiplatelets may become necessary due to interventions for coronary heart disease (CHD). Double antiplatelet therapy (DAPT) for at least 12 months is a routine treatment approach after percutaneous coronary interventions (PCI). However, in patients using OAT for AF, DAPT is recommended only for a short period (up to 1 month) for patients with acute CHD undergoing PCI. In this context, DOAC should be preferred, whenever possible, over warfarin. Following a short period of triple therapy, treatment with DOAC and P2Y_12_ inhibitor should be continued for 12 months [1]. In selected patients, at very high thrombotic risk, triple therapy can sometimes be prolonged or single antiplatelet therapy continued along with DOAC for a longer period. This approach may seem appropriate due to the nature of thrombotic event or type of stent used. However, it may also become problematic and cause harm more than benefit, if the treatment approach is not based on a flexible, individualized approach for older patients. It should be kept in mind that antiplatelet treatment should be no longer continued if a patient is already using an OAT and has a stable CHD (which refers to acute coronary syndrome or PCI more than 12 months ago) or peripheral artery disease (PAD) (PAD requiring an intervention more than 1 month ago) [113, 114].

Inappropriate medication use and polypharmacy, drug–drug and drug–disease interactions are common and should be taken into account in older patients treated with OAT [115]. Especially, warfarin users should be closely monitored for several drug, supplement and food interactions. NSAIDs should be avoided to reduce bleeding risk. Patients using concomitant OAT and antiplatelet therapy or corticosteroids should be given proton-pump inhibitors to avoid gastrointestinal bleedings [116, 117]. Main drug–drug interactions of DOACs involve P-glycoprotein (P-gp) and CYP3A4 CYP2Y2 competition and inhibition. Major contraindications for increased anticoagulant effect include concomitant use of anti-fungal drugs (itraconazole, ketoconazole, voriconazole, posaconazole) and quinidine virtually for all DOACs. Clarythromicin and erythromycin increase the anticoagulant effect in DOAC-treated patients, as well as amiodarone and dronedarone do in patients receiving dabigatran, rivaroxaban and edoxaban: dose-adjustment or use of a different DOAC should be considered in these circumstances [34]. There is evidence that concurrent use of amiodarone, rifampin, fluconazole and phenytoin in patients taking DOACs is associated with increased risk of MB compared with use of DOACs alone [118]. Furthermore, supplements commonly used in older adults (like ginkgo biloba) can cause serious bleedings when used concomitantly with OAT [114]. There is increasing evidence of several other drug interactions with potential clinical significance, including antineoplastic and antiepileptic drugs, of common use in older patients [2]. Therefore, use of DOACs in older patients mandate a careful evaluation of co-medications to optimize therapy and select the most appropriate drug and dose [34, 114].

## Conclusions and outlook

As the world population grows in terms of size and age, the challenges related to diagnosis and management of AF are also expected to rise and pose healthcare practitioners the multifaceted challenges of complexity [119]. These include—far beyond organ-related medicine, multimorbidity and/or chronological age—aspects related to age-related physiological changes, physical and cognitive functional impairment as well as multidimensional frailty—a surrogate marker of biological age.

While geriatric medicine provides a series of screening methods targeting AF-influencing domains such as daily functions, social status, cognitive performance, mood and nutrition, evidence on the systematic comanaged adoption and interpretation of the CGA in older AF patients is lacking. This would be the necessary next step to improve recommendations and guidelines in the near future and the basis for implementation of CGA in clinical routine. CGA-based instruments, indeed, help to optimizing the patient-centered decisions in older AF patients. To inform this evidence and improve recommendations and guidelines in the near future, further systematic research is necessary to link results of the CGA to patient outcomes, treatment benefits/risk and clinical decision making.

## Expert opinion box

- The large majority of AF patients are old-old and oldest-old. Recent DOACs RCTs, which demonstrated net clinical benefit over warfarin, mostly excluded oldest-old, frail and cognitively impaired individuals.
- Although RCTs and observational studies demonstrated that DOACs provide clinical benefit over warfarin in the majority of older patients, there is scant evidence of net clinical benefit in frail, disabled and severely cognitive impaired patients
- Due to the lack of evidence, physicians' attitudes prevail, with high risk of undertreatment and, at the same time, of futile therapy. Decisions regarding AF treatment in older adults require a more specific and holistic approach; CGA, MPI and appropriate frailty tools may assist physicians in clinical decision making
- The risk of fall should not be considered a contraindication to the use of DOACs. High bleeding risk should not be interpreted as a contraindication to the use of OAT, but should direct physicians to assess and manage modifiable bleeding risk factors.
- Inappropriate DOAC dosing, especially inappropriate “reduced” dosing is highly prevalent in older adults, but real-life studies do not provide evidence of a net clinical benefit of this strategy. Use of antiplatelet therapy as an alternative to OAT is not recommended, due to lower stroke prevention and high bleeding rates.
- Opportunistic screening of AF in older population is cost-effective and should be performed by pulse palpation or ECG rhythm strip. Annual events (like influenza vaccination) can be a good opportunity for AF screening in older adults.

## Data Availability

Does not apply.

## Code availability

Does not apply.
